# The role of suprasegmental cues in perception of sentences with linguistic ambiguity under informational masking

**DOI:** 10.3389/fnins.2025.1655467

**Published:** 2025-09-18

**Authors:** Jing Shen, Gayle DeDe

**Affiliations:** Department of Communication Sciences and Disorders, College of Public Health, Temple University, Philadelphia, PA, United States

**Keywords:** suprasegmental cues, speech perception in noise, pupillometry, listening effort, linguistic complexity, informational masking

## Abstract

**Introduction:**

Real-life communication contains rich and informative suprasegmental cues, such as variations in intensity, duration, and fundamental frequency. Although suprasegmental information is an essential component of spoken communication, we know little about its role in speech perception in the presence of background masker. Building on literature showing that suprasegmental cues facilitate the processing of spoken sentences with linguistic ambiguity, we addressed two questions in the present study. First, does the facilitative effect of suprasegmental cues on speech recognition interact with the amount of informational masking in speech maskers? Second, how do listeners use suprasegmental and lexico-semantic cues when listening to linguistically ambiguous sentences masked by competing speech maskers?

**Methods:**

We collected both offline performance data (recognition accuracy) and online processing effort data (pupil dilation) from 37 young adults with age-typical hearing. The speech material consisted of 15 sets of temporarily ambiguous early closure sentences, each with two suprasegmental conditions (facilitative vs. neutral) and two lexico-semantic conditions (transitive vs. intransitive subordinate verb). These sentences were embedded in original and time-reversed two-talker speech maskers differing in the amount of informational masking.

**Results:**

Recognition accuracy was higher with facilitative suprasegmental cues, particularly in maskers with less informational masking, as well as with facilitative lexico-semantic cues. Listeners expended greater processing effort throughout the sentence when suprasegmental cues were neutral, especially under more adverse conditions (i.e., stronger informational masking or greater linguistic ambiguity).

**Discussion:**

This study makes multiple contributions to the literature. First, the recognition accuracy data showed that informational masking and linguistic ambiguity interact with suprasegmental effects: these adverse conditions reduce the benefit of facilitative suprasegmental cues for speech recognition. Second, under stronger informational masking and greater linguistic ambiguity, the absence of facilitative suprasegmental cues increased effort during online speech processing. Third, we found that facilitative suprasegmental cues improved immediate recall of segmental information (i.e., words) in speech perception in speech maskers. Finally, our accuracy and effort data demonstrate the importance of using both offline and online measures of speech processing, as each reveals different aspects of the dynamic process of speech perception under adverse conditions.

## Introduction

For the past few decades, research on speech perception has provided substantial evidence on perception of segmental information (i.e., phoneme and word recognition). As building blocks of speech, acoustic cues on the segmental level are considered the essential components for speech perception. Unsurprisingly, they are also at the center stage for clinical testing as most speech perception tests are focused on recognition accuracy of words and phonemes. However, clinicians and researchers have come to realize the limitations of only using simple and neutral speech material (e.g., short sentence with low complexity) to measure speech perception outcomes, because they often fall short in predicting real-life communication experiences (Beechey, 2022).

Considering how we communicate in real life scenarios, it is evident that processing of speech requires far more than simple word recognition. Real life communication contains rich and informative suprasegmental cues, such as changes in intensity, duration, and fundamental frequency (Karimi-Boroujeni et al., 2023), that support the use of speech in social interactions. They scaffold top-down word recognition for efficient speech analysis and memory in real-time (Carlson, 2009; DeDe, 2010; Kjelgaard and Speer, 1999; Steinhauer et al., 1999); they also serve essential roles for conveying pragmatics, affective states, and attitudes (Dávila-Montero et al., 2021; Hellbernd and Sammler, 2016; Scherer, 1986). Taken together, this suggests missed suprasegmental cues will lead to interrupted processing of linguistic and social information, elevated processing error and effort, and communication breakdowns and withdrawal from social interactions. Not surprisingly, suprasegmental cues have been used as essential features in analyzing, modeling, and synthesizing real-life conversations by communication engineers for machine-enhanced social interactions (Dávila-Montero et al., 2021). In contrast, sentence-level clinical speech audiometry measures do not include facilitative acoustic cues that extend across phonemes and words. This narrow focus on perception of segmental cues (i.e., phonemes and words) in audiology research can only be a barrier to advancing clinical assessment and treatment. A critical step in this work, therefore, is to understand the role of suprasegmentals in speech understanding in adverse environments.

### The role of suprasegmental cues in language processing

Focusing on their roles in language processing in quiet environments, suprasegmental cues are recognized at an early processing stage as they can be identified without the fine-grained analysis necessary to recognize phonetic segments (Beach, 1991; Carroll and Slowiaczek, 1987; Kjelgaard and Speer, 1999). Indeed, listeners’ expectations of the structure of upcoming speech are influenced by suprasegmental cues as the speech signal is unfolding (DeDe, 2010; Engelhardt et al., 2010; Weber et al., 2006). Thus, the role of these cues during speech perception in quiet can be viewed as providing a structure/frame for forming processing units (Kjelgaard and Speer, 1999; Slowiaczek, 1981). Consider the phrase “*While the parents danced…*” The next word could be part of the same clause (e.g., “… *danced the tango*”) or begin a new clause (e.g., “… *the children sang*”). Linguistically, there are reasons to predict either continuation, but listeners are more likely to predict a new clause if “*danced*” is pronounced with a falling pitch contour and a lengthened duration (i.e., more sustained intensity). These facilitative suprasegmental cues, which support accurate sentence interpretation, can improve recall of spoken messages, support syntactic processing, and reduce processing effort for listeners with normal hearing in quiet listening conditions (DeDe, 2010; Engelhardt et al., 2010; Kjelgaard and Speer, 1999).

The role of suprasegmental cues in speech processing has also been investigated in the context of the interaction between these acoustic cues and the linguistic (e.g., lexical, syntactic) cues. For instance, Kjelgaard and Speer (1999) studied the interaction between suprasegmental cues and syntactic structure in comprehension of early and late closure sentences with both online and offline measures (i.e., cross modal naming, acceptability judgment). They found the suprasegmentals could influence sentence parsing by triggering an early closure interpretation despite other syntactic constraints. Using transitive and intransitive garden-path sentences (e.g., “When the parents danced/watched the child…”), DeDe (2010) measured self-paced listening times to examine the interaction between suprasegmentals and transitivity of the subordinate verbs during syntactic ambiguity resolution. The results showed suprasegmentals influenced sentence parsing as soon as they are available, and the effect was modulated by lexical properties of the subordinate verbs. This evidence has been replicated using other online measures such as eye-movement (Weber et al., 2006) and Event Related Potential (ERP, Steinhauer et al., 1999). This body of literature converges to demonstrate the online nature of the suprasegmental effects and suggests that these acoustic cues can interact with linguistic properties to modulate language processing in real time.

### Effects of suprasegmental cues in adverse environments

While the effect of suprasegmental cues on language processing has been demonstrated by psycholinguistic research, suprasegmental cues are often considered to be redundant with speech perception in quiet, because there is little challenge to necessitate the use of suprasegmental cues when the environment is favorable. Real life communication, however, often happens in crowded public places with background masker. When listeners encounter the perceptual challenges of background masker and have difficulty perceiving segmental cues (Lacroix and Harris, 1979; Stelmachowicz et al., 1990), they may rely on suprasegmental cues to deduce sentence structure that aids in the prediction of words, as well as “fill in the gaps” with missing segmental information. The processing of suprasegmental cues is also believed to occur at an early stage of language analysis and can be used without fine-grained analysis of phonetic segments (Kjelgaard and Speer, 1999). This is also consistent with ERP evidence indicating the perception of suprasegmental cues is pre-attentive in language processing (Leitman et al., 2009; Zora and Csépe, 2021). On this account, speech material with absent (i.e., neutral) suprasegmental cues might be significantly more difficult to recognize in more challenging acoustic environments. Theoretically, this hypothesis gains support from the Ease of Language Understanding model (Rönnberg et al., 2013). The ELU suggests that listeners heavily rely on top-down cognitive mechanisms when lexical items are not easily parsed from the speech stream due to acoustic degradation. The model, however, is largely based on speech intelligibility data with low-context sentences (e.g., “A large size stocking is hard to sell.”) and describes neither the processing that occurs between word recognition and understanding of the message nor the interaction between processing of segmental and suprasegmental cues in noisy environments (Wingfield et al., 2015). Built on this rationale, it is worth noting that while suprasegmental cues can be extracted automatically, integrating them into sentence parsing under challenging conditions may still draw on cognitive resources such as working memory, consistent with the explicit reappraisal outlined in the ELU. In this study, we aimed to test the hypothesis that suprasegmental cues are one of the mechanisms that aid top-down processing in masker, when masker makes lexical access more effortful. This *cue reliance hypothesis* suggests that the perceptual challenges from the environment would result in greater reliance on suprasegmental cues, which predicts facilitative suprasegmentals should improve recognition and reduce effort.

In parallel, there is evidence that perceptual challenges from background masker have a negative downstream effect on language processing. These effects have been explained by a limited capacity model in which mental resources are shared between perceptual and cognitive processes (Dillon, 1995; Picou and Ricketts, 2014; Rabbitt, 1991). On this account, when more mental resources are needed to cope with effects of a degraded acoustic signal and/or strong perceptual interference at the phoneme/word level, fewer resources are available for using suprasegmental cues to support linguistic processing, which leads to reduced suprasegmental effects on speech perception outcomes. The Framework for Understanding Effortful Listening (Pichora-Fuller et al., 2016) formalizes this shared resource account to address effort and fatigue in complex listening environments. Built on this rationale, the competing *resource limitation hypothesis* suggests that the perceptual challenges in the more adverse masker conditions can make recognition of segmental information effortful, resulting in the reduced use of suprasegmental cues in speech processing.

In addition to recognition and comprehension accuracy measures, previous studies have used eye-movement and pupillometry data to demonstrate the online effects of suprasegmental cues (Engelhardt et al., 2010; Weber et al., 2006). This type of online processing measure is particularly important for revealing the effects of suprasegmentals in speech perception because of the fast, moment-by-moment nature of sentence processing. In other words, suprasegmental cues are processed in an online manner incorporated with the linguistic context, while the sentence is unfolding. As offline performance measures (e.g., response accuracy) only reflects the end result of comprehension, pupil dilation can reveal momentary effort during processing, which can increase with perceptual novelty and cognitive load (e.g., Shen et al., 2022). In the present study, we used the measure of pupil dilation, which has been frequently used by recent research to indicate processing effort during speech perception under adverse conditions (Winn et al., 2018; Zekveld et al., 2018). In previous research, both eye movement and pupillometry measures are typically analyzed within a time window that is immediately after the critical point in the sentence. On the other hand, the processing effort due to degraded lexical and suprasegmental cues is likely to last longer and impact language processing beyond those transient moments. These online changes can provide the critical information in understanding the overall impact of suprasegmental cues in the context of adverse listening environments. In this study, we used a new analysis approach to examine the online effect from suprasegmental cues with informational masking without the restriction of a time window. Specifically, the Generalized Additive Mixed Models (GAMMs, Sóskuthy, 2021) were used to reveal the effort changes across the duration of the sentence. GAMMs are an extension of generalized additive models that allow for the incorporation of random effects to model correlated responses within participants and are well suited to handle real-time non-linear relationships between pupil dilation response and stimuli/conditions.

### Speech processing challenges due to informational masking

Real-life communication often happens in crowed public places with other people talking in the background. The speech masker in this scenario creates the well-known “speech-on-speech” effect, with its challenge attributed to informational masking (Freyman et al., 2004; Kidd and Colburn, 2017; Kidd et al., 2008). Informational masking is thought to stem from interacting perceptual and cognitive factors beyond the peripheral mechanism of spectral overlap between target and masker (i.e., energetic masking, Kidd and Colburn, 2017). For instance, a 2-talker speech masker has the same amount of energetic masking as unintelligible time-reversed maskers, but 2-talker maskers are perceptually similar to the target speech because they contain both segmental and suprasegmental cues. As a result, it may be more difficult to segregate the target from 2-talker maskers (Brungart et al., 2009; Calandruccio et al., 2017). We know that these perceptual and cognitive mechanisms in informational masking render speech perception more challenging. While previous research has focused on the impact of informational masking on segmental cues (i.e., phonemes and words), it is unknown whether informational masking affects how suprasegmental cues are used in speech perception. Practically, real-life communication often, but not always, includes facilitative suprasegmental cues. Knowing how these cues interact with informational masking can lead to more ecologically valid clinical outcome measures and interventions. For instance, if the suprasegmental cues play important roles in noisy environments with speech maskers, audiological assessments should include these cues in testing materials to measure individuals’ responses to weaker or missing suprasegmentals in masker. More targeted signal processing algorithms in hearing devices can be developed to alleviate the negative impact from hearing loss to the perception and use of suprasegmental cues.

Built on the *cue reliance* and *resource limitation* hypotheses proposed in the last section, our first objective was to test how informational masking from intelligible speech maskers affect the role of suprasegmental cues in speech recognition by younger listeners with typical hearing, as indicated by speech recognition accuracy and processing effort. The *cue reliance hypothesis* predicts that the listeners will rely more heavily on the suprasegmental cues with stronger informational masking from the speech masker, as recognition of segmental cues becomes more challenging. On the other hand, the *resource limitation hypothesis* would argue that the use of suprasegmental cues in speech processing requires cognitive resources and can be negatively impacted by the informational masking from the intelligible speech masker. Specifically, when separation of the target speech from the intelligible maskers becomes cognitively effortful, it can reduce the mental resource for using the suprasegmental cues to process the content of speech efficiently. As a result, following the r*esource limitation hypothesis*, the suprasegmental cues are predicted to have a weaker effect in the masker condition with stronger informational masking.

### Interaction between suprasegmental and lexico-semantic cues

While the psycholinguistic literature has demonstrated the online effects of suprasegmental cues on speech processing, the interaction between suprasegmental and lexical cues can potentially be altered by the increased challenge with lexical access in speech maskers (Hoen et al., 2007). When there is no background masker, listeners typically rely on the lexico-semantic status of the subordinate verb (e.g., transitive or intransitive) to parse the sentence structure and process the content in real time, even if the suprasegmental cues are absent or weak. For example, consider the sentence “when the parents danced/watched, the children sang a song.” When listeners hear the intransitive verb “danced,” they know there is likely a prosodic boundary immediately after this verb, followed by the start of the main clause. In contrast, when listeners hear the transitive verb “watched” they anticipate a direct object and assume the main clause does not start until later. Thus, listeners are more likely to interpret “the children” as the object of “watch.” As a result, they must reconcile the misanalysis when they hear the main verb in the clause “*sang* a song.” Put another way, all of the lexico-semantic cues support the correct interpretation of the sentence in the “danced” condition, while the cues conflict with the correct interpretation in the “watched” condition. Therefore, the transitive verb condition (i.e., “watched”) should in general cost more effort in online processing than the intransitive one, with or without suprasegmental cues.

We know that lexical access can become more challenging with speech maskers (Jesse and Helfer, 2019). As a result, the listeners may have to rely more heavily on the suprasegmental cues to parse the sentence in the more ambiguous transitive verb condition, which can lead to a stronger suprasegmental effect. This view is consistent with the *cue reliance hypothesis.* Alternatively, in the transitive verb condition, a larger amount of cognitive resource may be deployed to access the lexicons and to resolve the syntactic ambiguity, leaving little resource for using suprasegmental cues. This is predicted by the competing *resource limitation hypothesis*, which predicts a weaker suprasegmental effect in the transitive verb than in the intransitive verb condition. The second objective of the present study was to examine whether and how suprasegmental effects are modulated by lexico-semantic conditions as measured by recognition accuracy and processing effort in speech maskers.

## Methods

### Participants

Thirty-seven younger adults (*M* age = 21.43, range 18 to 32 years) were recruited from Temple University and the surrounding community. Eighteen participants were tested in the original speech masker condition; nineteen were tested in the reversed speech condition. Twenty-seven of participants identified as female, 9 as male, and 1 as nonbinary. Twenty-six of participants identified as White, 7 as Black/African American, 1 as Asian, 3 as more than one race. All participants were native English speakers with age-typical hearing (air-conduction threshold ≤ 20 dB HL across 0.5, 1, 2, 4 kHz). Three participants had slightly elevated thresholds of 25 dB HL at 250 and 8 k Hz. They were free of neurological, otological, and uncorrected visual disorders by self-report. Participants were either paid or awarded course credit for their time.

### Material

Speech material consists of sentences drawn from previous psycholinguistic studies that focused on the role of suprasegmental cues on sentence processing (DeDe, 2010). Fifteen sets of temporarily ambiguous early closure sentences were used (e.g., “While the parents danced/watched the child sang a song…”). These items are ambiguous at the word “the child,” because a word in that sentence position could be the object of the subordinate verb “dance/watch” or the subject of the main clause. As noted above, both verb transitivity and semantic constraints support the interpretation that “the child” is the subject of the main clause in the intransitive condition. In contrast, both transitivity and semantic constraints initially lead the listener to predict that “the child” is the object of “watched.” The sentence is disambiguated once it becomes clear that “the child” is the subject of “sang.” Ambiguous sentences were chosen because they afford the opportunity to observe whether suprasegmental cues influence the interpretation and effortfulness of processing. Each item is produced both with a relatively flat, albeit natural, suprasegmental contour and with a suprasegmental contour that supports one interpretation (subordinate clause for the ambiguous sentences, so-called early closure ambiguities). For example, in the facilitative condition, “danced” is pronounced with increased intensity and duration and a falling f0 to denote an “auditory comma” in “When the parents danced, the child…” Materials were normed (DeDe, 2010) to ensure that prosodic contours elicited the intended interpretations and that flat prosodies were judged to be natural.

There are 60 sentences in total (15 items recorded in two suprasegmental and two lexico-semantic conditions). Table 1 contains a set of 4 sentences (one item) as an example. To prevent listeners from anticipating sentence structures, filler items were developed with suprasegmental cues consistent with parenthetical constructions (e.g., “the girl, answered the boy, is late”), so-called late closure structure (e.g., “While the parents danced the tango, the child…”), and unrelated items. Filler and experimental items were combined and divided into 4 lists of 95 sentences, with item and suprasegmental condition counterbalanced across lists. Sentence order was pseudorandomized so that no more than two consecutive trials contain sentences from the same stimulus set.

**Table 1:** Sample item of the sentence stimuli.

	Intransitive verb condition	Transitive verb condition
Neutral suprasegmental condition	When the parents danced the child sang a song with her grandmother.	When the parents watched the child sang a song with her grandmother.
Facilitative suprasegmental condition	When the parents danced, the child sang a song with her grandmother.	When the parents watched, the child sang a song with her grandmother.

Stimuli were recorded in a sound attenuated booth by a female talker of Standard American English who has background in phonetics/phonology. Spoken sentence duration was controlled across conditions. Recorded sentences were manually segmented and trimmed to create individual sound files. All items were band-filtered to 80-10 k Hz and root-mean-square normalized before being embedded in maskers. To examine informational masking from an intelligible speech masker, a 2-talker speech masker and its time-reversed version, previously developed by Shen and Souza (2018), were used. The masker condition was a between-participant variable, and a fixed target-to-masker-ratio (TMR) of −5 dB was used for both masker conditions to control for energetic masking across conditions. Presentation level was set to be 65 dB SPL at the baseline and then amplified for individuals, using the National Acoustics Laboratories-Revised (NAL-R) linear prescriptive formula (Byrne et al., 2001) with individual thresholds averaged across ears. This amplification was implemented to facilitate comparison between data from younger and older adults (which are not reported in this article).

### Procedure

Participants completed the study in 4 weekly visits, with one stimulus list per visit. The task was repeating back the sentence immediately after they listen to it. Lists began with 5 practice trials. During testing, participants were seated in a dimly lit double-walled sound booth in front of an LCD monitor. Auditory stimuli were presented binaurally over Sennheiser HD-25 headphones. Pupil diameter data were collected using an Eyelink 1,000 plus eye-tracker in remote mode with head support. The sampling rate was 1,000 Hz and the left eye was tracked when possible. The distance between the eye and the screen was set to 58 cm. The screen color was set to gray to avoid outer limits of the range of pupil diameter (Shen, 2021). The luminance was measured as 37 lux at eye position. The experiment was implemented with a customized program using Eyelink Toolbox (Cornelissen et al., 2002) in MATLAB. First, a red fixation “X” appeared at the center of the screen. The auditory stimulus started playing after 2000-ms of silence for the eyes to stabilize on the “X” to obtain baseline pupil measurement. There was a 2000-ms silent retention period after the offset of the stimulus. After the participant responded by repeating back the sentence, the trial was terminated by the experimenter. A grey box was displayed at the center of the screen for 6,000-ms to allow the pupil to return to baseline before the next trial. The participant was instructed to blink and rest their eyes when the grey box was in display. Pupil diameter data were recorded continuously throughout the session, with the data file tagged with time stamps that were synchronized to each visual and auditory event.

### Data processing and analysis

For both recognition accuracy and pupillometry data, we built separate models to examine how the effects of suprasegmental cues on accuracy and effort are modulated by masker conditions and lexico-semantic conditions, respectively. This approach allows us to test theoretically motivated interactions without imposing an artificial 3-way dependency. The 2-way interaction models would also provide clearer insights for interpretation than 3-way interaction models. Therefore, the data were collapsed across lexico-semantic conditions in the suprasegmental by masker analysis, and across masker conditions in the suprasegmental by lexico-semantic analysis.

#### Speech recognition accuracy

Speech recognition accuracy data were analyzed using mixed effects logistic regression with package lme4 (Bates et al., 2014) in R version 4.0.3 (R Core Team, 2021). A base model (*model 1*) that included only masker condition (original masker vs. reversed masker) was compared to a model (*model 2*) including suprasegmental cues (facilitative vs. neutral), to test the main effects of both masker and suprasegmentals. Following the recommended practice of using mixed effects models (Meteyard and Davies, 2020), the primary models were built based on our study objectives, with fixed effects for both variables (masker and suprasegmentals) added first, followed by interaction between these variables (*model 2*). In all models, we included fixed factors of testing block order, and allowed for by-participant and by-item (i.e., sentence) random intercepts. We also tested all models with by-participant and by-item random slopes, but they failed to converge (Barr et al., 2013). For accuracy data, we used the model fit statistics of *p*-values from chi-square tests to identify the best fitting model.

A second model was built based on the same approach to examine the main effects and interactions of suprasegmental cues and lexico-semantic conditions, collapsed over masker conditions.

#### Pupil response

Pupil diameter data were pre-processed using R (Version 4.0.3) with GazeR library (Geller et al., 2020). As pupil diameter is shown to be altered by fixation location (Gagl et al., 2011; Hayes and Petrov, 2016), a center area of the screen was defined by ± 8° horizontal and ± 6° vertical to obtain pupil alteration rate less than 5% (Hayes and Petrov, 2016). Pupil diameter data were removed from further analysis when the fixation location was outside of this area, resulting in removal of 1.25% of data points. For the rest of pupil diameter data, gaze position on the screen was also included in the models to control for this variability. To control for the participant-level and trial-level pupil diameter variability before speech processing, two normalization steps were implemented in the analysis. First, individual’s maximum and minimum pupil diameters (measured within each trial) were used to calculate the range of pupil dilation, which was defined as the difference between these two values. The pupil diameter data were normalized by finding its ratio to individual’s dilation range. Secondly, the baseline pupil size data were calculated based on mean pupil size recorded during the 1,000 ms time period immediately before the onset of sentence. Pupil dilation was normalized relative to baseline pupil size of each trial by subtraction. The pupil dilation data were downsampled to 10 Hz (by aggregating data every 100 ms) before statistical analysis.

Pupil response data (trial by trial, between 0 and 5,000 ms relative to sentence onset) were first analyzed using a Generalized Additive Mixed Model (van Rij et al., 2019; Wieling, 2018) to examine whether the magnitude of pupil dilation throughout each trial was affected by suprasegmental and masker conditions, while controlling for the trend of pupil dilation over time. GAMMs are an extension of mixed-effects regression models that allow for the incorporation of random effects to model correlated responses within participants. This is a newer approach that has been used recently for the analysis of pupillometric data (van Rij et al., 2019), with several strengths over traditionally utilized Linear Mixed Models and Growth Curve Analyses. The key strength is that the GAMM method can handle non-linear random effects which will allow us to investigate any potential non-linear relationships between pupil dilation response to changes in suprasegmental cues and SNR. Additionally, GAMM allows us to include an autoregressive error term to account for autocorrelational errors in the data, reducing Type I error. Our models incorporate an autoregressive error model at lag = 1 (AR1) with an autocorrelation coefficient *rho = 0.98* estimated from a base model without the inclusion of AR1. By including the autocorrelation coefficient, the GAMM models can better account for the temporal autocorrelation across time, leading to more reliable inferences about the fixed effects in the models.

To test our first hypothesis, a series of models were constructed. First, a base model (*model 0*) was built that included linear and smooth (i.e., non-linear) terms of masker condition to examine its independent effect on speech perception, a second model added in the effect of suprasegmental conditions in linear and smooth terms (*model 1*), and a third model included an interaction between masker and suprasegmental conditions (*model 2*). The linear term is used for testing the difference in slope of pupil dilation change over time. The smooth term is for testing the differences in the shape of pupil dilation contour across time. For example, if pupil dilation contour in one condition increases more rapidly during a particular time window but converges later in time with the contours in other conditions, this effect can be captured by a smooth term. All the models used 10 knots for smooth functions (Sóskuthy, 2021), which allows for a maximum of 9 basis functions. To account for variation across participants and items, we included random effects for participant and item in all models. All models included a controlled factor smooth of gaze position on screen, factor smooths that model the pupil size over time for participant interacting with suprasegmental and masker conditions, a factor smooth that models the pupil size over time per participant, as well as a factor smooth that models the individual variation over time by item. The formula for *model 2* is appended below.

pupil ~ Suprasegmental * Masker.
+ s(x_eye_gaze, y_eye_gaze).
+ s(timeonset).
+ s(timeonset, by = Suprasegmental, k = 10, bs = “cr”).
+ s(timeonset, by = Masker, k = 10, bs = “cr”).
+ s(timeonset, by = Suprasegmental * Masker, k = 10, bs = “cr”).
+ s(timeonset, Participant, bs = “fs,” k = 10, m = 1).
+ s(timeonset, Participant, by = Suprasegmental, bs = “fs,” k = 10, m = 1).
+ s(timeonset, Participant, by = Masker, bs = “fs,” k = 10, m = 1).
+ s(timeonset, Item, bs = “fs,” k = 10, m = 1).

To demonstrate the non-linear effects due to suprasegmental effects in different masker conditions, we built another GAMM model to test and visualize the non-linear differences across all four combinations of suprasegmental and masker conditions (Wieling, 2018). Specifically, difference plots were created to identify time windows of significant differences in pupil size over time for each of the suprasegmental contrasts by masker combinations. For these pupil dilation data, we used AIC comparison for model selections because this method is more appropriate for models built using fREML method, which optimize the fitting of smooth terms that are of particular interest in this study (Pedersen et al., 2019; Wood et al., 2016). We interpret the significant findings based on the summary results of best fitting models, which are presented in the Results section.

The same approach was used to build a second set of models to test the second hypothesis of the study that the effect of suprasegmental cues on processing effort is modulated by the lexico-semantic conditions. All models were built in R version 4.0.3 (R Core Team, 2021) using packages *mgcv* and *itsadug* (van Rij et al., 2017; Wood and Wood, 2015; Wood, 2011).

## Results

### Recognition accuracy data

**Figure 1:**
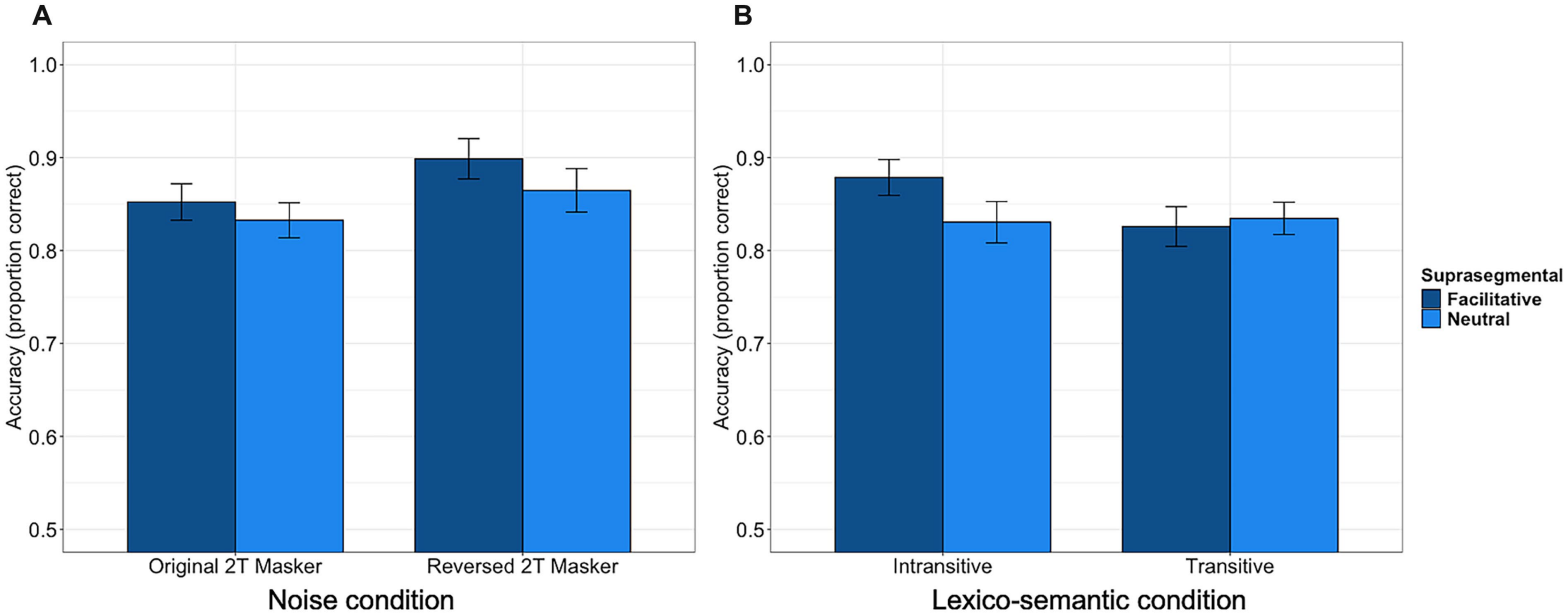
Panel **A** (Left) and **B** (Right): Recognition Accuracy.

In the study, we first examined the effects of suprasegmental cues and informational masking on speech recognition accuracy and processing effort (as measured by pupil dilation). Using a fixed SNR of −5 dB, the overall intelligibility levels were high (above 80%) in both maskers. Recognition accuracy data are reported in Figure 1. For accuracy data, the model comparison results showed the factor of suprasegmental cues, [χ^2^(6) = 24.605, *p* < 0.001], as well as the interaction between suprasegmental cues and informational masking, [χ^2^(7) = 6.678, *p* = 0.009], significantly improved model fit. The results from the final model (*model 2*) revealed significantly higher accuracy with facilitative suprasegmental cues (*β* = −0.426, *p* < 0.001) and with less informational masking (*β* = −0.617, *p* = 0.02). The model further showed a significant interaction between suprasegmental cues and informational masking (*β* = 0.283, *p* = 0.009). Specifically, the accuracy difference between neutral and facilitative suprasegmental cues was stronger in the reversed speech masker (i.e., less informational masking), as compared to in the original speech masker (see Figure 1 panel A).

For our second objective of examining the interaction between suprasegmental cues and linguistic conditions, the model comparison results suggested both suprasegmental cues [χ^2^(6) = 24.629, *p* < 0.001] and its interaction with linguistic condition [χ^2^(7) = 8.522, *p* = 0.003] significantly improved model fit. The results from the final model (*model 2*) showed significantly lower accuracy with neutral suprasegmental cues (*β* = −0.438, *p* < 0.001) and transitive verbs (*β* = −0.325, *p* < 0.001). The model further showed a significant interaction between suprasegmental and lexico-semantic cues (*β* = 0.319, *p* = 0.003). Sentences with intransitive verbs and facilitative suprasegmental cues were associated with significantly more accurate responses than any other conditions.

### Pupil dilation data

**Figure 2:**
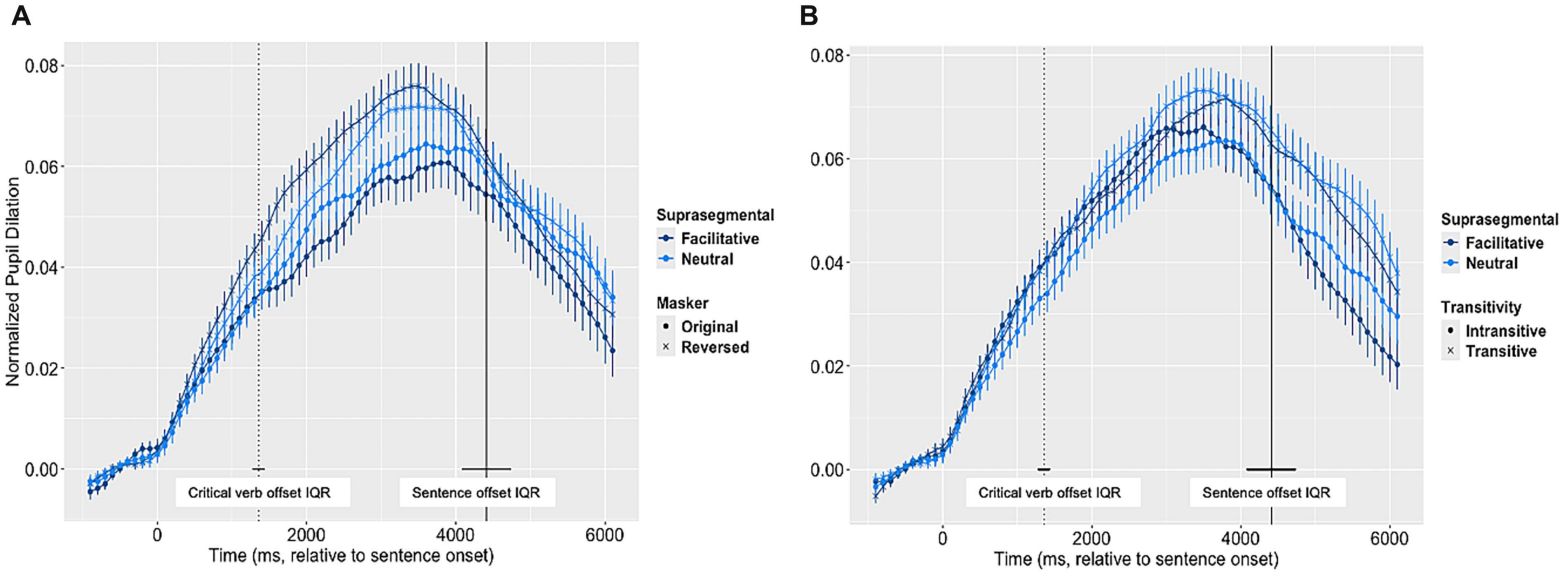
Pupil dilation trajectories. Panel **A** (Left): Suprasegmental and Masker conditions; and **B** (Right): Suprasegmental and Transitivity (Lexico-semantic) conditions. The vertical dotted lines indicate the mean offset of the critical verbs, and the vertical solid lines indicate the mean sentence offset, with interquartile ranges (IQR) marked using horizontal lines. The error bar represents + 1 standard error.

Figure 2 reports pupil dilation trajectory data for each of the two objectives. For our first objective, we ran a series of GAMM models to examine the main effect of masker (i.e., with or without informational masking) on pupil size over time (*model 0*), the inclusion of both masker and suprasegmental cues as main effects (*model 1*), and lastly examining interactions between masker and suprasegmentals (*model 2*). We found that the addition of suprasegmental cues into the base model decreased the AIC (AIC_difference_ = 47082.02) and increased the explained deviance of the model. Same was found for the inclusion of the interaction (AIC_difference_ = 15007.42). Therefore, we chose *model 2* as our final model. The summary of all model output is found in Table 2.

**Table 2:** Summary of the Generalized Additive Mixed Models on normalized pupil dilation data: suprasegmental cues and informational masking.

	Model 0: Base model				Model 1: Full model				Model 2: Interactions			
	Estimate	SE	*t*-value	Pr (>|*t*|)	Estimate	SE	*t*-value	Pr (>|*t*|)	Estimate	SE	*t*-value	Pr (>|*t*|)
Parametric coefficients												
(Intercept)	0.030	0.014	2.107	**0.035**	0.029	0.014	2.121	**0.034**	0.033	0.014	2.368	**0.017**
Informational masking	0.007	0.019	0.378	0.705	0.002	0.019	0.116	0.907	−0.006	0.020	−0.340	0.733
Suprasegmental	---	---	---	---	0.006	0.003	1.762	0.078	−0.025	0.006	−4.223	**<0.001**
Suprasegmental x informational masking	---	---	---	---	---	---	---	---	0.063	0.001	94.831	**<0.001**
	Edf	Ref.df	*F*-value	*p*-value	Edf	Ref.df	*F*-value	*p*-value	Edf	Ref.df	*F*-value	*p*-value
Smooth terms												
s(x, y) [gaze position]	28.889	28.999	34334.589	**<0.001**	28.895	28.999	34210.000	**<0.001**	28.895	28.999	34124.804	**<0.001**
s(time)	7.440	7.562	7.093	**<0.001**	6.579	6.759	7.940	**<0.001**	6.620	6.798	6.012	**<0.001**
s(time): informational masking	5.713	6.213	1.984	0.059	6.553	7.002	3.706	**<0.001**	6.524	6.971	5.023	**<0.001**
s(time): suprasegmental	---	---	---	---	7.962	8.164	1.571	0.107	7.617	7.911	7.786	**<0.001**
s(time): suprasegmental x informational masking	---	---	---	---	---	---	---	---	7.130	7.893	188.502	**<0.001**
s(time, subject)	299.513	369.000	28.096	**<0.001**	295.249	369.000	40,550	**<0.001**	296.905	369.000	27.529	**<0.001**
s(time, subject): informational masking	71.291	199.000	165.645	**<0.001**	74.507	199.000	240,500	**<0.001**	72.675	200.000	150.782	**<0.001**
s(time, subject):suprasegmental	---	---	---	---	355.483	370.000	295.3	**<0.001**	355.378	370.000	285.427	**<0.001**
s(time, item)	138.489	149.000	414.897	**<0.001**	138.493	149.000	400.8	**<0.001**	138.498	149.000	397.335	**<0.001**
	R^2^ (adjusted): 0.2				R^2^ (adjusted): 0.204				R^2^ (adjusted): 0.205			
	Explained deviance: 16.1%				Explained deviance: 16.4%				Explained deviance: 16.5%			

We report parametric coefficients, effective degrees of freedom (Edf), reference degrees of freedom (Ref.df), F-values, and *p*-values for the smooth terms and random effects. The values in bold reflect significance at least the *p* < 0.05 level.

The parametric effects from the final GAMM model first showed an effect of suprasegmental cues. Pupil dilation was overall larger with facilitative suprasegmental cues as compared to neutral cues (*β* = −0.025, *p* < 0.001), suggesting that the facilitative suprasegmental cues exerted a greater processing demand. Similar to the accuracy data, the pupillometry data showed an interaction between suprasegmental and masker conditions, with a suprasegmental effect in the opposite direction in the presence of the intelligible speech masker as compared to the reversed speech masker (interaction between suprasegmental and masker condition: *β* = 0.063, *p* < 0.001), which is in contrast with the result from accuracy data.

**Figure 3:**
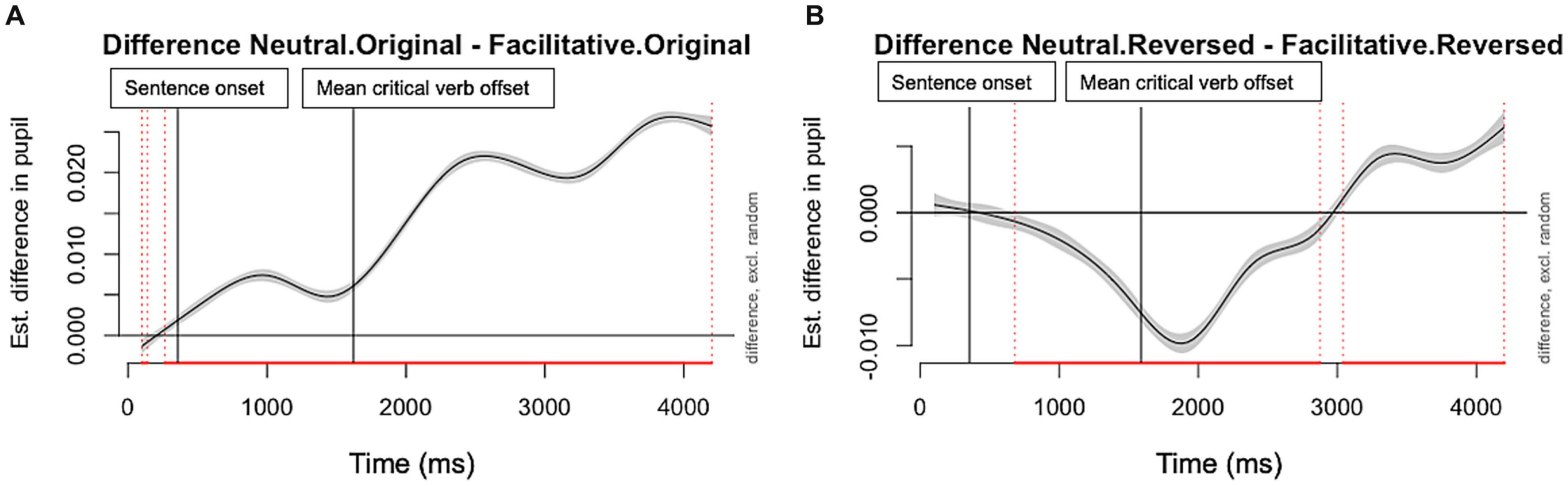
Pupil dilation difference trajectories (between neutral and facilitative suprasegmental conditions). Panel **A** (Left): Original Speech Masker; and **B** (Right): Reversed Speech Masker. The red lines on the X axis and the dotted vertical lines indicate the time frame with significant differences between the two suprasegmental conditions. The regions falling below the zero line indicate increased pupil dilation in the facilitative condition compared to the neutral condition. The vertical lines indicate the critical timepoints in the sentence.

**Figure 4:**
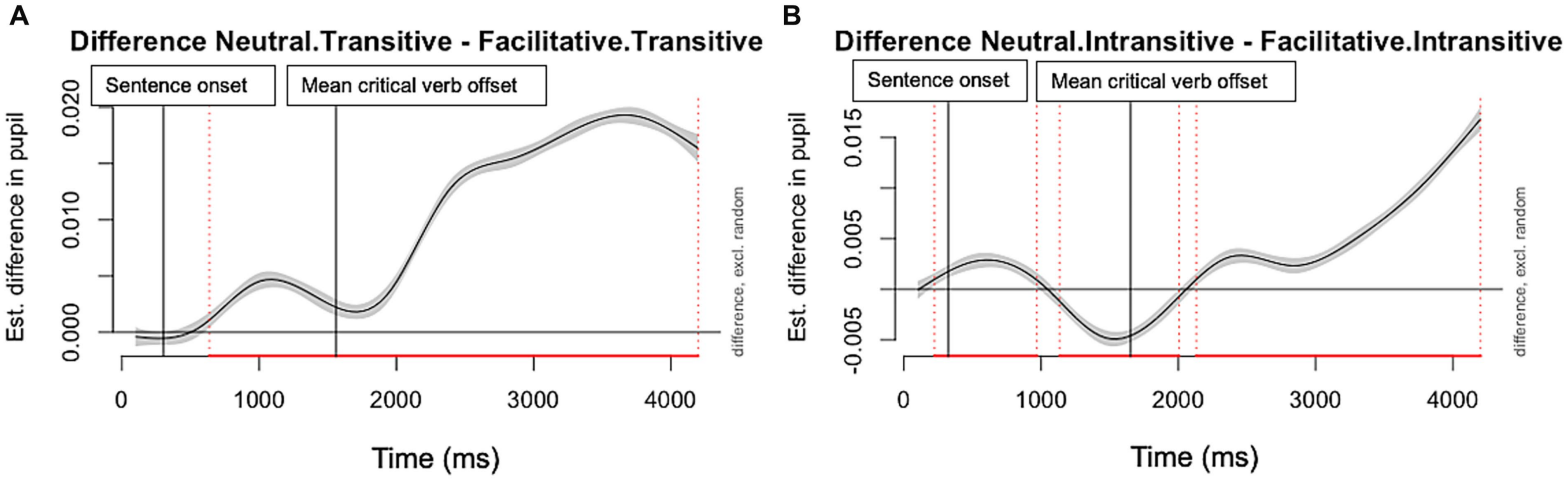
Pupil dilation difference trajectories (between neutral and facilitative suprasegmental conditions). Panel **A** (Left): Transitive Verb; and **B** (Right): Intransitive Verb. The regions falling below the zero line indicate increased pupil dilation in the facilitative condition compared to the neutral condition. The red lines on the X axis and the dotted vertical lines indicate the time frame with significant differences between the two suprasegmental conditions. The vertical lines indicate the critical timepoints in the sentence.

One of the advantages of using GAMM is the ability to examine the difference of pupil dilation between conditions while the speech is unfolding. We were interested in whether the interaction between suprasegmental cues and informational masking manifests itself differently over the time course of the sentence. We used difference plots to visualize the model estimates of the difference in pupil size over time (smooth terms) between neutral and facilitative suprasegmental conditions in the two masker conditions (Figures 3, 4). The solid lines in each difference plot show us the difference between the two non-linear smoothed trajectories between suprasegmental conditions in each of the two masker conditions, while the shaded areas represent the 95% confidence intervals. Inspection of the data showed a complicated pattern of results. Figure 3, panel A shows that, when the masker was intelligible (original speech masker), the neutral condition was more resource demanding than the facilitative condition across the entire sentence. In contrast, when the masker offered less informational masking (Figure 3, panel B), the facilitative condition was initially more demanding. This difference reversed direction around 1,700 ms after the onset of the sentence. This time point corresponds to the effect on pupil dilation (with 400 ms delay from the acoustic signal) from the end of the subordinate clause (i.e., “danced/watched,” 1,360 ms from sentence onset averaged across sentences). This pattern of pupil response time locked to the critical verb suggests the facilitative suprasegmental cues around the verb provided cues that reduced processing effort in the facilitative condition during online processing.

For the second objective of examining the interaction between the suprasegmental cues and the lexico-semantic factor, a series of GAMM models were built using a similar structure as those in the first objective. First model included the main effect of lexico-semantic cues (i.e., transitive vs. intransitive verbs) on pupil size over time (*model 0*), the inclusion of both lexico-semantic and suprasegmental cues as main effects (*model 1*), and lastly examining interactions between lexico-semantic and suprasegmental cues (*model 2*). We found that the addition of suprasegmental cues into the base model decreased the AIC (AIC_difference_ = 58124.44) and increased the explained deviance of the model. Same was found for the inclusion of the interaction (AIC_difference_ = 1525.00). Therefore, we chose *model 2* as our final model. The summary of all model output is found in Table 3.

**Table 3:** Summary of the Generalized Additive Mixed Models on normalized pupil dilation data: Suprasegmental cues and lexico-semantic conditions.

	Model 0: Base model				Model 1: Full model				Model 2: Interactions			
	Estimate	SE	*t*-value	Pr (>|*t*|)	Estimate	SE	*t*-value	Pr (>|*t*|)	Estimate	SE	*t*-value	Pr (>|*t*|)
Parametric coefficients												
(Intercept)	0.034	0.010	3.301	**<0.001**	0.030	0.010	2.945	**0.003**	0.032	0.010	3.123	**0.001**
Lexico-semantic	0.005	0.004	1.209	0.226	0.004	0.004	0.973	0.330	−0.001	0.004	−0.123	0.901
Suprasegmental	---	---	---	---	0.006	0.003	1.794	0.072	−0.001	0.003	0.442	0.658
Suprasegmental x lexico-semantic	---	---	---	---	---	---	---	---	0.009	0.0001	58.921	**<0.001**
	Edf	Ref.df	*F*-value	*p*-value	Edf	Ref.df	*F*-value	*p*-value	Edf	Ref.df	*F*-value	*p*-value
Smooth terms												
s(x, y) [gaze position]	28.897	28.999	35611.124	**<0.001**	28.90	28.999	35680.181	**<0.001**	28.901	28.999	35734.496	**<0.001**
s (time)	6.978	7.136	8.035	**<0.001**	4.49	4.727	7.768	**<0.001**	4.984	5.216	8.153	**<0.001**
s(time): lexico-semantic	7.315	7.620	1.948	0.069	7.42	7.711	2.165	**0.044**	7.544	7.840	3.203	**0.002**
s(time): suprasegmental	---	---	---	---	8.08	8.268	1.573	0.105	8.334	8.494	2.305	**0.016**
s (time): suprasegmental x lexico-semantic	---	---	---	---	---	---	---	---	8.642	8.954	148.591	**<0.001**
s (time, subject)	352.016	370.000	1643.944	**0.010**	352.88	370.000	4718.448	**<0.001**	352.545	369.000	7993.040	**<0.001**
s (time, subject): lexico-semantic	357.675	370.000	408.787	**<0.001**	357.41	370.000	410.929	**<0.001**	357.326	370.000	416.920	**<0.001**
s (time, subject):suprasegmental	---	---	---	---	356.13	370.000	314.265	**<0.001**	355.950	370.000	314.644	**<0.001**
s (time, item)	138.607	150.000	431.907	**<0.001**	138.78	150.000	421.982	**<0.001**	138.732	149.000	417.196	**<0.001**
	R^2^ (adjusted): 0.241				R^2^ (adjusted): 0.248				R^2^ (adjusted): 0.248			
	Explained deviance: 19.5%				Explained deviance: 20.1%				Explained deviance: 20.1%			

We report parametric coefficients, effective degrees of freedom (Edf), reference degrees of freedom (Ref.df), F-values, and *p*-values for the smooth terms and random effects. The values in bold reflect significance at least the *p* < 0.05 level.

The results from the parametric terms in model 2 showed non-significant main effects of suprasegmental and lexico-semantic cues and a significant interaction between suprasegmental and lexico-semantic cues (*β* = 0.009, *p* < 0.001). The pupil dilation was larger with neutral suprasegmental cues than facilitative cues, and the difference is larger with the sentences that have transitive verb than those with intransitive verb. The difference smooth term for the suprasegmental and lexico-semantic interaction was also significant in the final model, *F*(8.95) = 148.59, *p* < 0.001, indicating the pupil dilation changes over time was significantly impacted by the interaction. Figure 4 illustrated pupil dilation difference trajectories between the neutral and facilitative suprasegmental conditions, in two lexico-semantic conditions, respectively. This visual inspection of the difference curves indicates, in the transitive verb condition (Figure 4, panel A), pupil dilation was larger throughout the trial when facilitative suprasegmental cues were missing. In the intransitive verb condition (Figure 4, panel B), the difference curve hovered around 0 until about 400 ms after the critical verb and became positive after that, indicating the facilitative suprasegmental cues around the verb decreased processing effort during online processing.

## Discussion

### Effects of suprasegmental cues modulated by informational masking

The present study investigated the effects of suprasegmental cues on speech recognition in speech maskers. Using recognition accuracy and pupil dilation as dependent measures, we first examined how availability of suprasegmental cues affects speech recognition with different amounts of informational masking. Recognition accuracy was higher with facilitative suprasegmental cues, and particularly in the time-reversed speech masker that poses less informational masking. This result supports the *resource limitation hypothesis* and demonstrates the negative impact of intelligible speech maskers on the use of suprasegmental cues. Literature has shown that the intrusive lexical information in the intelligible speech maskers can interfere with linguistic processing, resulting in lower comprehension accuracy (Hoen et al., 2007). Built on this evidence, the current study made two new contributions to the field. First, we demonstrated that the effect of informational masking reduced the benefit from suprasegmental cues in resolving linguistic ambiguity. This finding is consistent with the FUEL framework (Pichora-Fuller et al., 2016) in demonstrating the downstream effect of informational masking. According to the FUEL framework, our cognitive capacity is finite and is shared by multiple processes during speech processing in background masker. When informational masking requires listeners to direct attention to concentrate on one of the speech streams, this process competes for cognitive resources with the goal of understanding the meaning of speech. Listeners used suprasegmental cues to facilitate speech understanding in the time-reversed masker with less informational masking, but the extra cognitive load of strong informational masking limited the mental resources available for effective use of suprasegmental cues. Further, we demonstrated it in speech recognition accuracy as measured by immediate recall, although the recognition accuracy appears to only require perception of segmental cues (i.e., words) regardless of suprasegmentals. The finding that suprasegmental cues improve immediate recall of sentences suggests suprasegmental cues can release working memory capacity from the negative impact of informational masking on speech perception. This is because suprasegmental cues facilitate syntactic analysis of sentences and further enhance short-term memory encoding (Kjelgaard and Speer, 1999; Wingfield et al., 1989). This effect can be particularly strong when additional informational masking heavily taxes working memory (Rönnberg et al., 2013) and hinders memory encoding of complex sentences. From a clinical perspective, this result supports the potential of recognition accuracy as an outcome measure for assessing individual listeners’ use of suprasegmental cues in speech perception. Although the effect was small (<5% accuracy) in the current dataset with a sample of younger adults with normal hearing, the effect size may increase in a group of older adults with hearing loss who can perceive suprasegmentals and are likely to benefit more from them. Further, while the time-reversed 2-talker masker is a frequently used control condition that is known to have comparable amount of energetic masking to speech maskers (Kidd et al., 2016), it also poses increased forward masking (Rhebergen et al., 2005). This could potentially counteract the decreased informational masking and reduce the observable effects. These questions should be examined by future research.

The pupil dilation data, on the other hand, indicated a more complex pattern with listening effort changes during online sentence processing. While the suprasegmental effect was stronger in the reversed masker condition with the offline measure of recognition accuracy, its online effect as shown by pupil dilation was more consistent and sustained in the intelligible speech masker that has stronger informational masking. This was demonstrated by an elevated pupil dilation with neutral suprasegmental cues than the facilitative ones, across the full duration of the sentence (Figure 3, panel A). In contrast, in the unintelligible time-reversed masker, pupil dilation was more elevated with facilitative than neutral suprasegmental cues in the first 2,500-ms of the sentence (Figure 3, panel B). This increase in processing effort with facilitative suprasegmental cues in time-reversed masker could be explained by additional processing resources associated with adapting to the perceptual novelty of the reversed masker early in the online processing. Compared to the original 2-talker speech masker, the time-reversed masker is a novel acoustic stimulus which listeners do not typically encounter in real-life scenarios. Therefore, the listener may be more perceptually engaged by this masker, as indicated by the overall heightened pupil dilation than in the original masker condition (Figure 2, panel A; Shen et al., 2022). This adaptation effect could lead to a longer time course for the listener to orient to the facilitative suprasegmental cues and benefit from them. This eventually leads to the increased pupil dilation with facilitative than the neutral suprasegmental cues during the early time window in the reversed speech masker, which was not observed in the intelligible speech masker with strong informational masking. Overall, these differences between the two informational masking conditions demonstrated the importance of using the online measures of processing effort, as they can reveal different aspects of the dynamic process of speech perception that can be attributed to acoustic and linguistic factors while a sentence is unfolding (Shen et al., 2013; Winn and Teece, 2021; Smith and Winn, 2025).

### Interaction between suprasegmental and lexico-semantic cues

Regarding the second research question of whether suprasegmental effect interact with the lexico-semantic cues in sentences, speech recognition data suggested the suprasegmental cues are helpful with congruent lexico-semantic cues (i.e., subordinate verb being intransitive, such as “when the parents *danced*, the child…”). For this type of sentence, having facilitative suprasegmental cues can aid sentence processing with improved recognition in speech maskers. In contrast, when the sentence is more linguistically complex with a transitive verb as subordinate verb (e.g., “when the parents *watched*, the child…”), syntactic processing was challenging with lower recognition performance regardless of whether the suprasegmental cues was facilitative. With facilitative suprasegmentals, the listener must resolve the conflict between the lexico-semantic cue of having “the child” being the direct object of the verb “watched,” and the auditory comma after “watched” based on the suprasegmental information suggesting “the child” is the start of a clause. With neutral suprasegmental cues, although the sentence appears to be congruent with no auditory comma in “when the parents watched the child…,” the processing load become much higher once they hear “sing a song” when there is a syntactic violation that triggers reanalysis. These results demonstrated that the effect of suprasegmental cues is modulated by the linguistic complexity in speech recognition with speech maskers, with a stronger benefit in less complex and more congruent linguistic conditions. Importantly, the effect was evident in speech recognition measure, which indicates the effect of suprasegmental cues on recognition tasks that heavily involve working memory. This also supports the feasibility of including suprasegmental cues in clinical assessments with recognition measures.

The complex interaction between suprasegmental and lexico-semantic cues was also evident in the pupil dilation data (Figure 4). Critically, the GAMM analysis provided evidence showing the effect of suprasegmental cues was modulated by temporary ambiguity that stems from lexico-semantic cues on processing effort during online sentence processing. Although the suprasegmental effect was stronger with intransitive subordinate verb in the accuracy data, its impact during online sentence processing revealed a temporal pattern that tightly coupled with the moment-to-moment processing (Figure 4, panel B). As soon as the intransitive verb was heard without facilitative suprasegmental cues, the processing effort as measured by pupil dilation increased significantly as compared to when there were facilitative suprasegmental cues. After the listener successfully parsed the sentence structure with the lexico-semantic cues (i.e., the intransitive verb “danced” cannot carry the objective of “the child” and therefore this must be a subordinate clause), the processing effort in the neutral suprasegmental condition decreased. Towards the end of the sentences when integration and recall posed higher processing load, the neutral suprasegmental appeared to elicit higher effort than the facilitative one. This pattern was in contrast with the pupil dilation in the transitive verb condition, in which the neutral suprasegmental condition consistently prompted larger pupil dilation across the whole sentence. The effort was particularly heightened between 2,500 and 3,500 ms post sentence onset, indicating the more effortful reanalysis upon hearing “sing a song,” after the initial commitment to having “the child” as the object of “watched.” These results converge with the processing effort data from speech perception literature (DeDe, 2010; Kadem et al., 2020; Zellin et al., 2011) in demonstrating the negative impact of missing suprasegmental cues on processing load with ambiguous sentences in real-time processing.

### The insights from a combination of recognition accuracy and pupil dilation measures

Overall, the current study contributed to the literature by demonstrating the effects of suprasegmental cues on speech recognition in speech maskers with informational masking. Converging across the measures of recognition accuracy and processing effort, the results showed an interesting pattern that illuminates on the role of suprasegmental cues in the dynamic process of effortful listening with perceptual and linguistic challenges. On one hand, the online measure of processing effort supported the cue reliance hypothesis by showing facilitative suprasegmental cues reduce processing effort more strongly and consistently in more adverse conditions with either perceptual or linguistic challenges (i.e., stronger informational masking, more linguistic ambiguity). On the other hand, the offline measure of recognition accuracy appears to be consistent with the resource limitation hypothesis in showing a reduced suprasegmental benefit for sentence recognition in more adverse conditions. It is also worth noting that these results were observed with an overall recognition accuracy of 80–90% indicating a favorable task condition. These findings first demonstrate that the cue reliance and resource limitation accounts are not mutually exclusive in effortful listening scenarios, at least when task difficulty is manageable. Although relying on facilitative suprasegmental cues reduced processing effort under more adverse conditions, the recognition accuracy was only improved by suprasegmentals in the less adverse conditions when more cognitive resource is available to utilize these cues in the sentence repeat-back task that heavily involves short-term memory encoding and retrieval. These results align with the literature suggesting listening effort as measured by pupil dilation data provides new information in addition to recognition accuracy, as it reveals the dynamic process of language processing that is tightly coupled with the speech material (Winn and Teece, 2021). This finding also raises the question for future research that whether listeners can benefit more from suprasegmental cues in more adverse conditions, if instead of repeat back sentences, the offline task is comprehension, which more closely simulates the real-life communication.

## Data Availability

The raw data supporting the conclusions of this article will be made available by the authors, without undue reservation.
